# The platelet to lymphocyte ratio predicts overall survival better than the neutrophil to lymphocyte ratio in metastatic renal cell carcinoma

**DOI:** 10.3906/sag-2009-75

**Published:** 2021-04-30

**Authors:** Oktay Halit AKTEPE1, Gürkan GÜNER, Deniz Can GÜVEN, Taha Koray ŞAHİN, Fadime Sinem ARDIÇ, Deniz YÜCE, Şuayib YALÇIN, Mustafa ERMAN

**Affiliations:** 1 Department of Medical Oncology, Faculty of Medicine, Hacettepe University, Ankara Turkey; 2 Department of Internal Medicine, Faculty of Medicine, Hacettepe University, Ankara Turkey; 3 Department of Preventive Oncology, Faculty of Medicine, Hacettepe University, Ankara Turkey

**Keywords:** NLR, PLR, metastatic renal cell carcinoma, prognosis, tyrosine kinase inhibitors

## Abstract

**Background/aim:**

The prognostic values of systemic inflammatory markers, neutrophil to lymphocyte ratio (NLR), and platelet to lymphocyte ratio (PLR) on overall survival (OS) of metastatic renal cell carcinoma patients (mRCC) treated with tyrosine kinase inhibitors (TKI) remain unclear. Thus, the present study aimed to investigate the prognostic impact of these markers on OS of mRCC patients.

**Materials and methods:**

A total of 150 patients receiving TKIs were retrospectively analyzed. Progression-free survival and OS times were analyzed with the Kaplan–Meier method, and the log‐rank test was used for comparison. Univariable and multivariable Cox regression models evaluated the impact of NLR and PLR on OS of the patients. The receiver operating characteristic curve analysis determined that the optimal cut-off values of NL, and PLR in predicting OS were 2 and 204, respectively.

**Results:**

Patient with PLR > 204 had significantly lower median OS time than those with PLR ≤ 204 (14.6 months vs. 31.6 months, P < 0.001). While the univariate analyses showed that both NLR and PLR associated with OS (NLR: P = 0.002; PLR: P < 0.001), PLR, not NLR, was an independent determinant for OS in the multivariate analyses (Hazard Ratio: 2.535, 95% CI: 1.564-4.108, P < 0.001). Additionally, the presence of brain metastases and International Metastatic Renal Cell Carcinoma Database Consortium (IMDC) prognostic scoring system were identified as independent prognostic factors for OS (brain metastases: P = 0.040; IMDC: P < 0.001).

**Conclusion:**

The PLR is a readily and inexpensively obtained marker, which may predict OS in patients with mRCC treated with TKIs.

## 1. Introduction

Renal cell carcinoma (RCC) is one of the most commonly seen malignancies, accounting for almost 4% of oncological disease burden [1]. Distant metastases have been detected in %20-30 of patients with RCC at the presentation [2]. Tyrosine kinase inhibitors (TKI), including sunitinib or pazopanib, are widely used first-line treatment options for metastatic renal cell carcinoma (mRCC) patients, targeting the vascular structure of RCC [3]. Heng et al. published the most commonly used current prognostic scoring system, which included hemoglobin count, neutrophil count, platelet count, corrected serum calcium level, Karnofsky performance status, and time to treatment initiation < 1 year, to provide individualized patient care and clinical trials development [4]. According to the International Metastatic Renal Cell Carcinoma Database Consortium (IMDC), the treatment options of patients with mRCC are individualized [5]. However, it seems rational to add new prognostic determinants to this scoring system for better prognostication and treatment of patients with mRCC. 

RCC has been evaluated as a highly immune-infiltrated tumor harboring a plenteous amount of infiltrating lymphocytes [6]. It was shown that clear cell RCC has the highest cell based on a T cell infiltration score and an immune infiltration score among the 19 cancer types analyzed by The Cancer Genome Atlas research program [7]. The determinants of a systemic inflammatory response, such as neutrophil to lymphocyte ratio (NLR), platelet to lymphocyte ratio (PLR), and the Glasgow prognostic score were suggested as independent predictors for survival outcomes in various types of solid tumors, including RCC. However, taking the studies conducted with NLR and PLR levels into consideration, there are conflicting findings. In patients with mRCC, Gunduz et al. found no significant association between the PLR and OS, but Park et al. showed PLR as a significant indicator for both progression-free survival (PFS) and overall survival [8, 9]. Furthermore, while Pichler et al. demonstrated that NLR was not a significant predictor for cancer-specific survival in patients with RCC (HR: 1.59, P = 0.148), Keizman et al found that low NLR levels are associated with better OS (HR: 0.3, P = 0.043) [10,11]. Thus, in the present study, we aimed to provide further knowledge about the prognostic impact of the systemic inflammatory markers, NLR and PLR, on OS in patients with mRCC receiving TKIs.

## 2. Materials and methods

### 2.1. Patients

A total of 150 consecutive patients with mRCC treated with TKIs between 2008 and 2019 at the Department Of Medical Oncology Unit of a Tertiary-Care Cancer Center were enrolled in the present retrospective-observational cohort study. The whole cohort was composed of patients with pathologically confirmed mRCC of any subtype, and those treated with TKIs (Pazopanib, and sunitinib) until progression, death, or unacceptable side effect. All the patients had cytokine treatment before initiation of TKIs. Patients who had an acute or chronic infection, autoimmune, and hematological diseases, chronic liver, renal diseases, and those receiving drugs were not included in the present study as these histories can affect the parameters measured in the complete blood count. The including patients had complete blood count data within 7 days before the initiation of TKIs, pazopanib, or sunitinib. Patients were stratified in three risk groups according to the IMDC scoring system (Favorable risk: no risk factor; intermediate risk: 1–2 risk factor; poor risk: > 3 risk factor). The demographic and clinicopathological characteristics variables of patients used in the study were gathered from our center’s electronic records. The study approval was obtained from the local ethics committee and all procedures in the present study have been conducted following the 1964 Helsinki declaration, and its later amendments.

### 2.2. Statistical analysis

Continuous variables were presented as median and interquartile range, and dichotomous variables were presented as percentages. Mann–Whitney U and Chi-square tests were used for comparison of continuous and categorical variables in the independent groups, respectively. NLR and PLR were calculated as the division of the absolute neutrophil count to the absolute lymphocyte count for NLR and division of the absolute platelet count to the absolute lymphocyte count for PLR. PFS was measured from the initiation of TKIs until progression and/or death. OS was measured from the initiation of TKIs until the last follow-up and/or death. Kaplan–Meier method was used for survival analyses, and a long-rank test was done to compare the differences between prognostic subgroups. The receiver operator characteristics (ROC) curves with Youden’s J index were plotted to determine the optimal cut-off values of NLR and PLR in predicting OS. The univariate and multivariate analyses were conducted using Cox proportional hazards regression models to define risk factors for OS. Multivariate analyses were performed using the variables with a P value of ≤ 0.25 in the univariate analyses. The statistical analyses in the present study were done using SPSS v25 (IBM Inc., Armonk, NY, USA) software, and a P < 0.05 was regarded as statistically significant.

## 3. Results

### 3.1. Patient characteristics stratified according to PLR values

**Figure 1:**
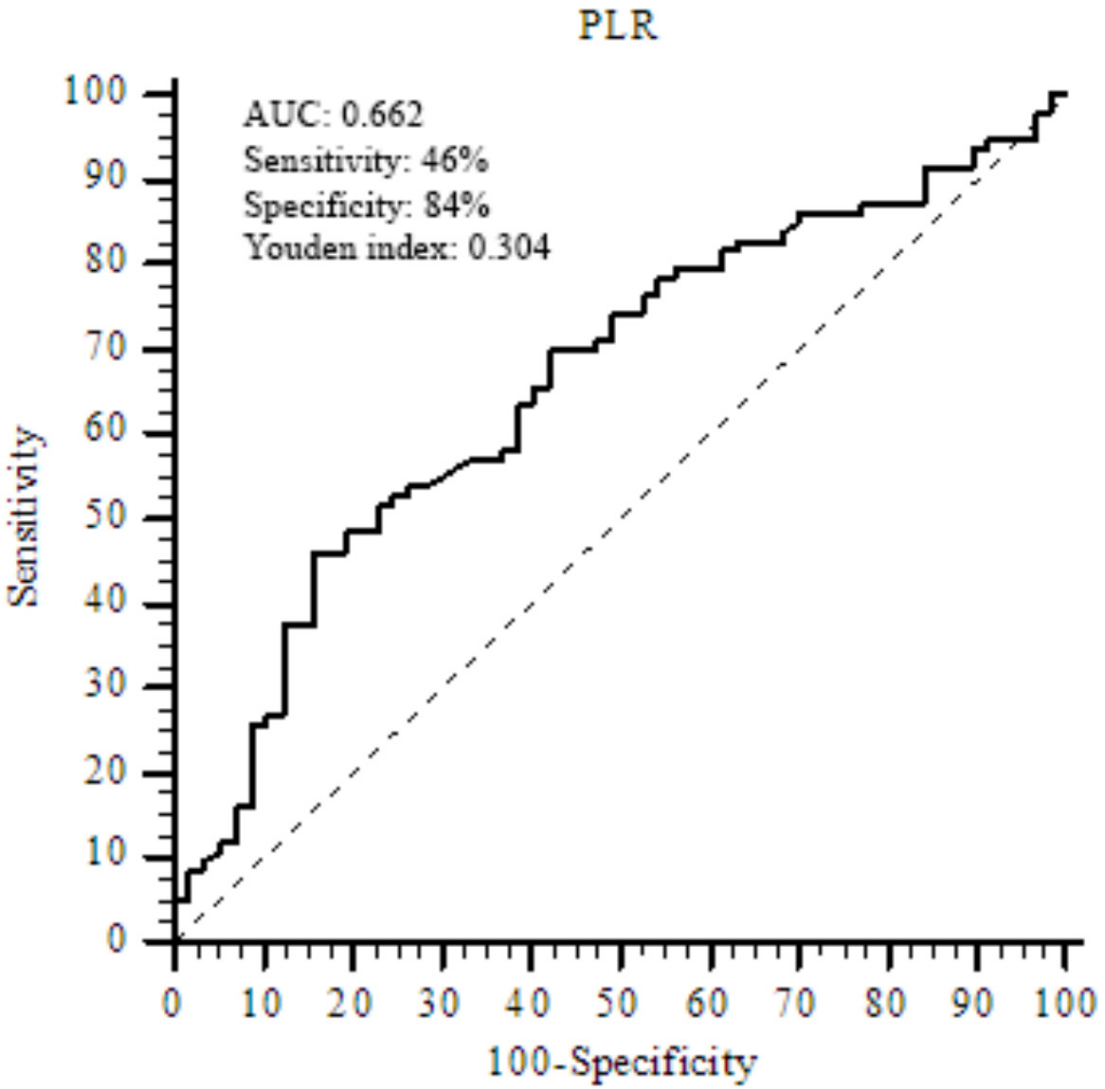
ROC curve showed that the PLR value of 204 was the optimal cut-off in predicting OS.

**Figure 2:**
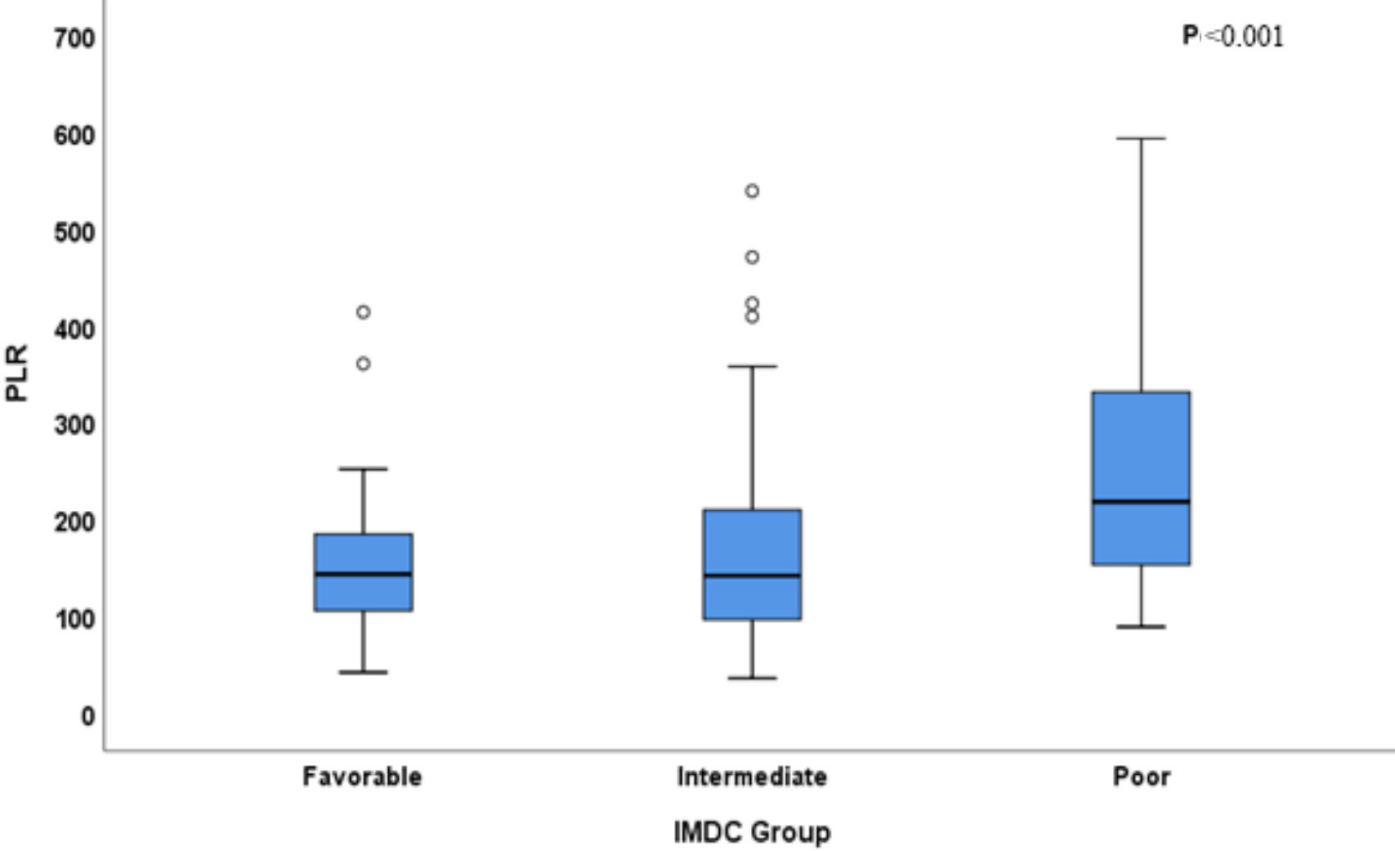
PLR values in IMDC subgroups.Kaplan–Meier estimates of OS in patients stratified according to IMDC risk groups.

Baseline demographic, clinical, histopathological characteristics of the patients stratified according to PLR values were summarized in Table 1. A total of 150 patients (male/female: 114/36) with a median age of 60 years, who were treated with TKIs for mRCC were enrolled after the exclusion of patient with acute or chronic infection (n = 8), autoimmune diseases (n = 2), hematological diseases (n = 6), chronic liver (n = 5), renal diseases (n = 6), and those with lost to follow up (n = 25). Completeness of the retrospectively collected data was 97% for histological subtype, 90% for tumor grade, and there was no missing data for remaining demographic and clinical parameters. Later lines of treatment choices included axitinib, everolimus, and nivolumab. Histologically, our study population was mostly composed of patients with clear cell RCC (78.5%). According to the IMDC scoring system, while more than half the patients were evaluated in the intermediate-risk group (57.3%), the percentages of patients with favorable, and poor-risk groups were 15.3% and 27.3%, respectively. By applying the ROC analysis, the NLR value of 2 was evaluated as the optimal cut-off point (AUC: 0.67; sensitivity: 63%; specificity: 77%, P = 0.0001), and PLR value of 204 evaluated as the optimal optimal cut-off point in predicting OS (AUC: 0.66; sensitivity: 46%; specificity: 84%, P = 0.0004) (Figure 1). While PLR values were not found statistically different between favorable and intermediate-risk group (Median PLR: 143 vs 141, respectively), the poor-risk group had significantly higher PLR values than the favorable and intermediate-risk group (Median PLR: 152, P < 0.001) (Figure 2). 

**Table 1:** Baseline patient characteristics stratified according to PLR cut-off.

Characteristics	Low PLR (≤ 204; n = 98)	High PLR (> 204; n = 52)	P value
Age (years)	59 (53–64)	61 (52–67)	0.235
Sex (%) Female Male	23.1 76.9	33.9 66.1	0.135
Histology (%) Clear cell Non-clear cell	80.8 19.2	75.9 24.1	0.463
Lung metastasis (%) Yes No	76 24	74.6 25.4	0.843
Liver metastasis (%) Yes No	18.3 81.7	30.5 69.5	0.073
Bone metastasis (%) Yes No	19.2 80.8	35.6 64.4	0.021
Brain metastasis (%) Yes No	4.8 95.2	3.4 96.6	1
Tumor grade (%) Grade I- II Grade III-IV	32.3 67.7	26.4 73.6	0.676
IMDC risk group (%) Favorable Intermediate Poor	18.3 65.4 16.3	6.8 50.8 42.4	0.001
Treatment type (%) Pazopanib Sunitinib	65.4 34.6	72.9 27.1	0.324
Second line treatment Axitinib Everolimus Nivolumab None	18.4 19.4 20.4 41.8	23.1 25 21.2 30.8	0.576
Third line treatment Axitinib Everolimus Nivolumab None	18.4 3.1 7.1 71.4	17.3 5.8 3.8 73.1	0.759
Forth line treatment Axitinib Everolimus Nivolumab None	3.1 2 3 91.8	3.8 - 1.9 94.2	0.955
OS (months)	31.6	14.6	< 0.001

### 3.2. The prognostic significance of NLR, and PLR for OS

**Figure 3:**
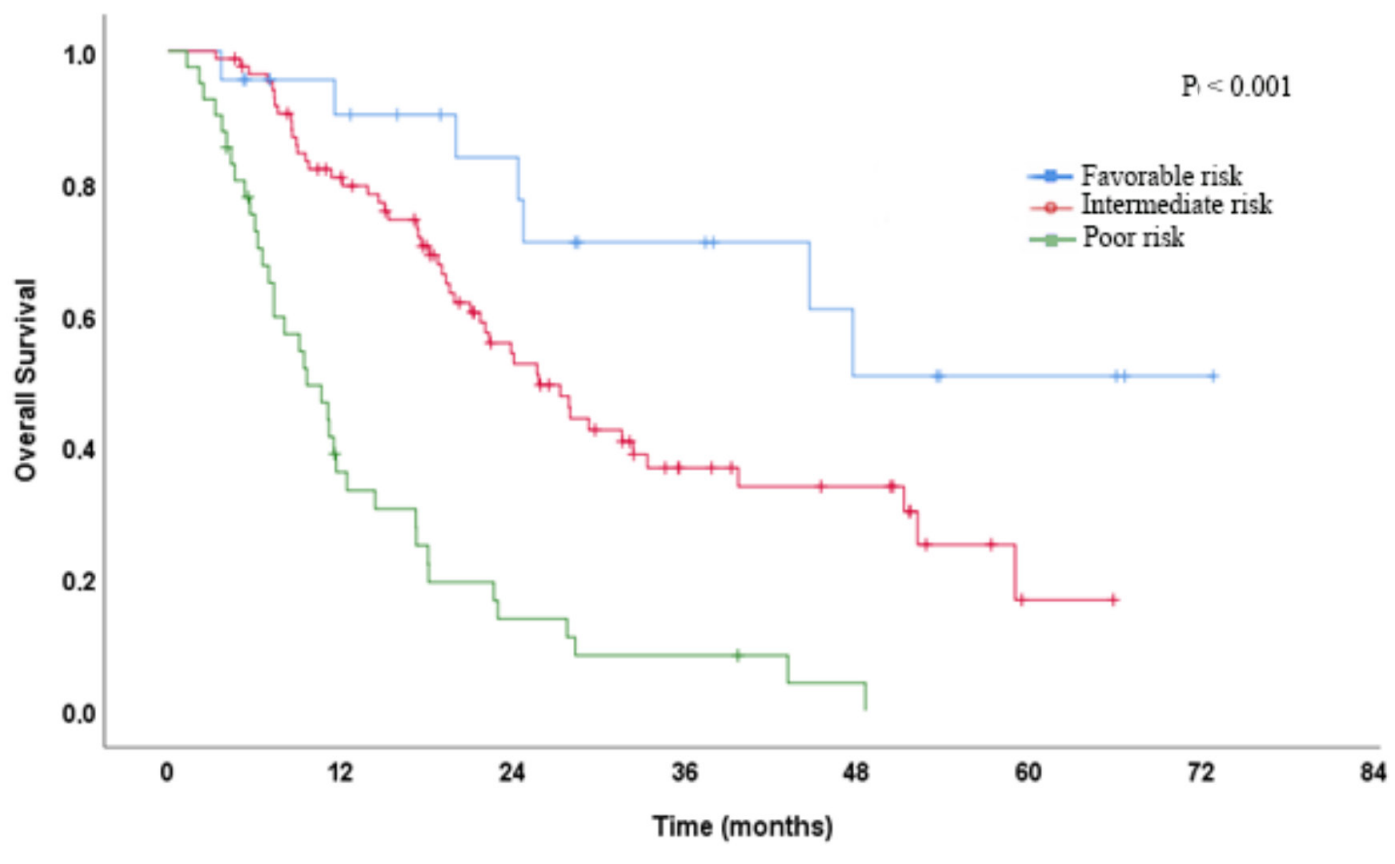
Kaplan–Meier estimates of OS in patients stratified according to IMDC risk groups.

**Figure 4:**
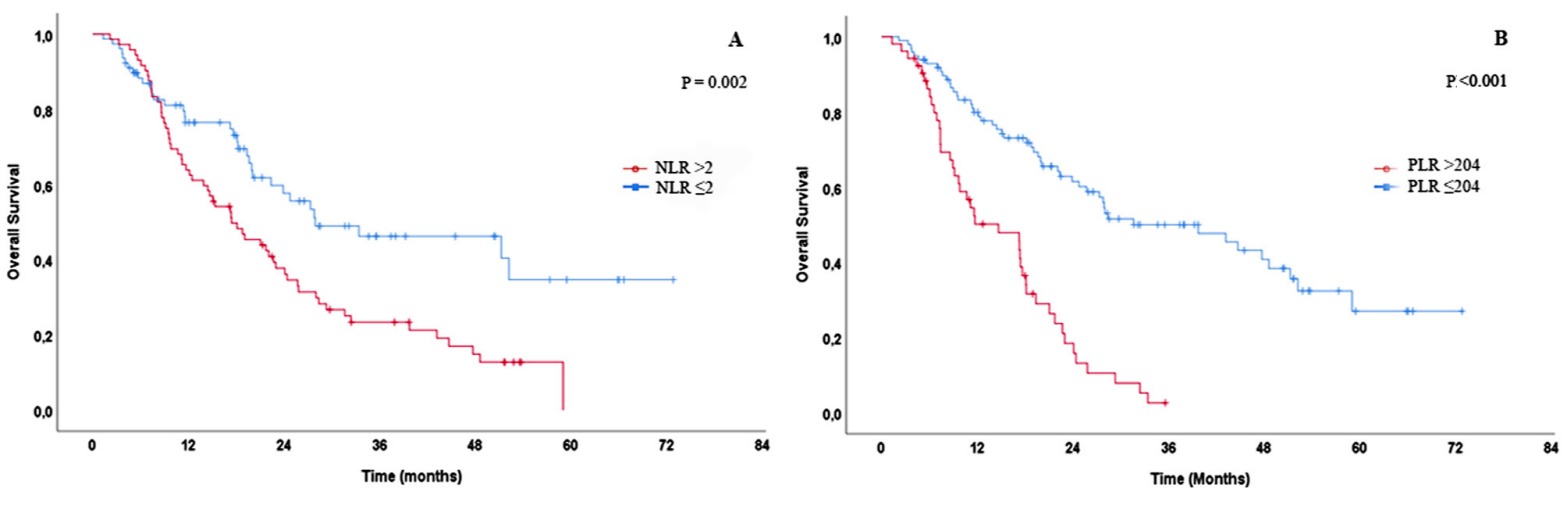
Kaplan-Meier estimates of OS in patients stratified according to NLR (A), and PLR (B) cut-off values.

Ninety-three (%62) patients died within the median follow-up time of 18 months (minimum-maximum: 1.3–72.8). The measured PFS and OS times of the whole population were 11 months (95% CI: 8.4–13.5), and 22.3 months (95% CI: 18.3–26.4). When we stratified the patients according to the IMDC scoring system, while the median OS time of patients with the favorable-risk group was not reached, on continuing follow-up, the median OS time of the patients with intermediate and poor-risk group was 25.7 months (95% CI: 20–31.4) and 9.6 months (95% CI: 5.9–13.3), respectively (Figure 3). Kaplan–Meier curves demonstrated that patients with higher NLR or PLR had significantly inferior OS times than those with lower NLR or PLR (NLR: 17.4 months vs. 27.9 months, P = 0.002; PLR: 14.6 months vs. 31.6 months, P < 0.001) (Figure 4A for NLR, Figure 4B for PLR). Taking the 3-year Kaplan–Meier estimate of survival into consideration, the survival rates of patients with higher or lower NLR and higher or lower PLR were 23% and 46% for NLR, while 3% and 50% for PLR, respectively. The presence of bone metastasis (P = 0.017), liver metastasis (P = 0.037), NLR > 2 (P = 0.002), PLR > 204 (P < 0.001), and IMDC scoring system (P < 0.001) were determined as significant prognosticators for shortened OS by the univariate Cox analysis (Table 2). However, multivariate Cox analyses, as shown in Table 3, PLR > 204, not NLR, were independent indicators in predicting OS (HR: 2.535, 95% CI: 1.564 - 4.108, P < 0.001). In addition to PLR, the other identified independent factors for OS were the presence of brain metastases (HR: 2.512, 95% CI: 1.041 - 6.066, P = 0.040) and the IMDC scoring system (P < 0.001).

**Table 2:** Univariate analyses demonstrating the associations between the variables and OS.

Variables	HR	95% CI for HR		P value
		Lower	Upper	
Age	1.012	0.994	1.030	0.198
Sex Male Female	reference 0.862	0.542	1.373	0.533
Tumor grade Grade I Grade II Grade III Grade IV	reference 0.463 0.466 0.646	0.107 0.110 0.154	2.007 1.972 2.706	0.418 0.303 0.299 0.358
Histology Clear cell Nonclear cell	reference 0.990	0.590	1.661	0.969
NLR ≤ 2 > 2	reference 1.937	1.268	2.960	0.002
PLR ≤ 204 > 204	reference 3.591	2.314	5.570	< 0.001
Lung metastases Absent Present	reference .897	0.552	1.460	0.660
Liver metastasesAbsentPresent	reference1.645	1.031	2.625	0.037
Bone metastasis Absent Present	reference 1.716	1.099	2.680	0.017
Brain metastasis Absent Present	reference 1.740	0.756	4.099	0.193
IMDC risk group Favorable Intermediate Poor	reference 2.338 8.105	1.053 3.543	5.188 18.539	< 0.001 0.037 < 0.001

**Table 3:** Multivariate analyses identifying the independent risk factors for prediction of OS.

Variables	HR	95% CI for HR		P value
		Lower	Upper	
Age	1.012	0.994	1.030	0.198
Liver metastases Absent Present	reference 1.157	0.706	1.896	0.563
Bone metastasis Absent Present	reference 1.279	0.796	2.056	0.309
Brain metastasis Absent Present	reference 2.512	1.041	6.066	0.040
NLR≤ 2 > 2	reference 1.274	0.811	2	0.294
PLR≤ 204 > 204	reference 2.535	1.564	4.108	< 0.001
IMDC risk group Favorable Intermediate Poor	reference 2.117 5.873	0.944 2.460	4.7481 4.020	0.069 < 0.001

## 4. Discussion

TKIs sunitinib and pazopanib are included in the first-line treatment options in patients with mRCC. However, it remains unclear which patient will respond better to the treatment. It seems quite necessary to develop new prognostic determinants to achieve better prognostic scoring system(s) because of the achievement of the insufficient concordance indexes of IMDC (0.74 to 0.82 for PFS; 0.68 to 0.89 for OS) [12]. There are emerging pieces of evidence that the markers of systemic inflammatory response including NLR and PLR may have a potential role in predicting OS of mRCC patients treated with TKIs. Our study revealed that while PLR > 204 was associated with OS shortening, NLR was not found as an independent predictor of OS.

NLR is an indicator of systemic inflammation response. Increased local and systemic response to tumor is associated with high NLR, which facilitates tumor invasion and metastasis by providing a suitable microenviroment [13]. Tumor-associated macrophages coming from monocytes have a role in resistance to antivascular endothelial growth factor (VEGF) directed therapy by causing neutrophilia via interleukin-6 secretion [14]. Lymphocytopenia and the depression of cell-mediated immunity caused by the chemokines and cytokines are other charecteristics of systemic inflammatory response [15]. It was reported that RCC with a Von Hippel-Lindau (VHL) mutation results in declined T lymphocyte count [16]. High NLR was shown to be associated with inferior outcomes in mRCC patients treated with TKIs in previous studies, and cut-off values of NLR ranged from 2 to 4 [17–19]. Our study showed that NLR value of 2 was the optimal cut-off point in predicting OS. However, NLR was not found as an independent predictor for OS in our multivariate analyses. The conflicting cut-off points in these studies may have resulted from the fact that the composition of histology (clear cell/non clear cell) and IMDC risk score groups may be different from one study to another. 

The elevated PLR has been shown as an independent determinant for survival outcomes of patients with diversified types of cancer including the most common ones. Wang et al. demonstrated that PLR is associated with poorer survival outcomes in patients with lung adenosquamous cancer (P = 0.001) [20]. PLR, not NLR, was found as an independent indicator in prediction for OS of patients with resectable colon cancer (HR: 1.97; P = 0.021) [21]. Krenn-Pilko et al. identified that breast cancer patients with elevated PLR had shortened OS than those with decreased PLR (HR: 1.92; P = 0.047) [22]. However, the exact impact of PLR on survival outcomes of RCC remains inconsistent. In the study conducted by Gunduz et al., they evaluated only the association between PLR, not NLR, survival outcomes of 100 mRCC patients, and showed that PLR was an independent predictor for PFS but not for OS (P = 0.029) [8]. However, Chrom et al. found that NLR ≥ 4, not PLR, was an independent predictor for OS (P < 0.001) [19]. In contrast to these findings, Park et al. analyzed the prognostic value of NLR and PLR along with the documented prognostic determinants in 63 mRCC patients treated with TKIs, and PLR > 150, not NLR, was associated with OS in their multivariate analyses (HR: 16.1; P = 0.001) [9]. Similar to the study by Park et al., we showed that PLR was an independent predictor for OS, and no significant association between NLR and OS was determined. The conflicting findings in these studies may have resulted from the inadequate measurement of the cut-off points by ROC analysis in one study than in the another. Furthermore, these cohort studies may be composed of heterogeneous patient populations who were stratified according to IMDC prognostic scoring systems.

The exact underlying influence mechanisms of altered PLR remain unclear. The study by Lu et al. revealed that chronic inflammation may take part in tumor formation and progression [23]. While RCC has an immunogenic nature, there is an inadequate knowledge in the literature about the roles of inflammatory pathways and immune cells in progression and immune escape mechanisms of RCC. VHL, a tumor suppressor gene, is involved in the carcinogenesis process of the vast majority of the patients with clear cell RCC [24]. Inactivated VHL results in an increased amount of hypoxia-inducible factor (HIF), which plays a role in the transcription of key genes related to tumor survival, including vascular endothelial growth factor (VEGF) [25]. HIF may take part in the creation of a tumor microenvironment rich in myeloid cells, neutrophils, and macrophages by stimulating chemokine production [26]. Increased interleukin-8 (IL-8) levels have been detected in serum samples of RCC cases, and IL-8 may influence the amount of neutrophil and platelet counts [27]. Thrombocytosis is a well-known predictor for worse oncologic outcomes in renal cell carcinoma [28]. VEGF, platelet-derived growth factor, hepatocyte growth factor, thrombospondins, and endostatin are secreted molecules from platelets, and they have a key role in angiogenesis and carcinogenesis process [29]. It was suggested that thrombocytosis participates in the progression of RCC by causing elevated interleukin-6 levels, which result in a sustained T helper type 2 cytokine response via stimulating macrophages and T-lymphocytes [30,31]. Lymphocytes are involved in the immune response of the host against the tumor, and it was shown that direct connection between tumor-infiltrating lymphocytes and tumor cells resulted in the cytolysis of tumor cells, diminished tumor burden, and create superior survival outcomes [32,33]. Nevertheless, it remains necessary to explain the exact association between the tumor cell and lymphocytes and platelets by future trials. 

The limitations of the present study include the retrospective nature of the study conducted at a single center. However, NLR and PLR were separately evaluated in predicting OS in most studies mentioned above. We analyzed the impact of both markers on OS with a relatively larger study population than the studies that were aimed to investigate the prognostic value of these markers in mRCC patients treated with TKIs. Furthermore, since our study mostly include patients with intermediate-risk (57.3%), future studies are needed to evaluate the prognostic role of PLR on mRCC in more homogeneous populations.

In summary, our results revealed that PLR > 204, the presence of liver metastases, and bone metastases were independent prognostic determinants in predicting OS of mRCC patients treated with TKIs. To create more accurate prognostication and better-personalized treatment in patients with mRCC, PLR might give an additive value to the current prognostic scoring system (IMDC). 

## Funding

The authors did not receive financial support for this research.
